# Novel role for anti-Müllerian hormone in the regulation of GnRH neuron excitability and hormone secretion

**DOI:** 10.1038/ncomms10055

**Published:** 2016-01-12

**Authors:** Irene Cimino, Filippo Casoni, Xinhuai Liu, Andrea Messina, Jyoti Parkash, Soazik P. Jamin, Sophie Catteau-Jonard, Francis Collier, Marc Baroncini, Didier Dewailly, Pascal Pigny, Mel Prescott, Rebecca Campbell, Allan E. Herbison, Vincent Prevot, Paolo Giacobini

**Affiliations:** 1 Inserm, Laboratory of Development and Plasticity of the Neuroendocrine Brain, Jean-Pierre Aubert Research Centre, U1172, Lille 59045, France; 2 University of Lille, FHU 1,000 Days for Health, School of Medicine and SFR DN2M, Lille 59045, France; 3 Centre for Neuroendocrinology and Department of Physiology, University of Otago School of Medical Sciences, Dunedin 9054, New Zealand; 4 Inserm U1085-IRSET, Université de Rennes 1, Rennes 35042, France; 5 Service de Gynécologie Endocrinienne et Médecine de la Reproduction, Hôpital Jeanne de Flandre, CHU de Lille, Lille 59037, France; 6 Laboratoire de Biochimie & Hormonologie, Centre de Biologie Pathologie, Centre Hospitalier Régional Universitaire, CHU de Lille, Lille 59037, France

## Abstract

Anti-Müllerian hormone (AMH) plays crucial roles in sexual differentiation and gonadal functions. However, the possible extragonadal effects of AMH on the hypothalamic–pituitary–gonadal axis remain unexplored. Here we demonstrate that a significant subset of GnRH neurons both in mice and humans express the AMH receptor, and that AMH potently activates the GnRH neuron firing in mice. Combining *in vivo* and *in vitro* experiments, we show that AMH increases GnRH-dependent LH pulsatility and secretion, supporting a central action of AMH on GnRH neurons. Increased LH pulsatility is an important pathophysiological feature in many cases of polycystic ovary syndrome (PCOS), the most common cause of female infertility, in which circulating AMH levels are also often elevated. However, the origin of this dysregulation remains unknown. Our findings raise the intriguing hypothesis that AMH-dependent regulation of GnRH release could be involved in the pathophysiology of fertility and could hold therapeutic potential for treating PCOS.

## Abstract (web summary)

Anti-Müllerian hormone (AMH) plays a role in sexual differentiation and gonadal function, but extra-gonadal effects of AMH are not known. Here Cimino *et al*. show that AMH activates a subset of gonadotrophin-releasing hormone (GnRH)-releasing neurons, contributing to luteinizing hormone secretion from the pituitary gland.

## Graphical abstract

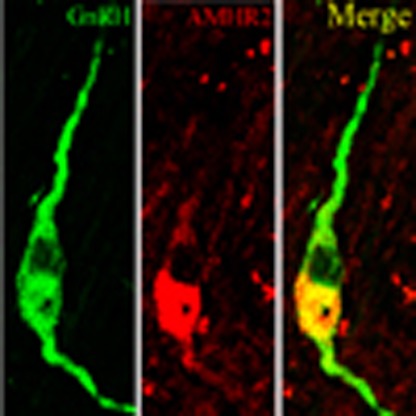

Reproduction in mammals is dependent on the function of specific neurons that secrete GnRH. These neurons send projections to the median eminence (ME) of the hypothalamus, which serves as an interface between the neural and peripheral endocrine systems. Here GnRH is released into the pituitary portal blood vessels for delivery to the anterior pituitary to elicit the secretion of luteinizing hormone (LH) and follicle-stimulating hormone (FSH)^1^. Alterations in the development of this system or in the secretion of GnRH are associated with a number of reproductive disorders including hypogonadotropic hypogonadism, hypothalamic amenorrhoea, hyperprolactinemia and polycystic ovary syndrome (PCOS)^2^,^3^. PCOS is a common complex endocrinopathy that occurs in up to 10% of women^4^,^5^. A hallmark of PCOS is increased LH concentrations and LH:FSH ratios^6^. Among the heterogeneity of symptoms in PCOS women, it is still not clear why the GnRH-induced LH level is altered.

In PCOS women, anti-Müllerian hormone (AMH) is suspected to play a significant role in causing anovulation due to its inhibitory influence on FSH that normally promotes follicular development from the small antral stage to ovulation stage^7^,^8^,^9^. Moreover, previous studies have shown that plasma AMH levels in PCOS patients are two- to threefold higher than in women with normal ovaries^10^,^11^, and that the severity of the PCOS phenotype correlates with AMH production, which is higher in anovulatory than in ovulatory PCOS patients^12^,^13^. AMH, also known as Müllerian-inhibiting substance, is a member of the transforming growth factor-β (TGF-β) superfamily^14^. It is a homodimeric glycoprotein with a molecular weight of 140 kDa, in which the two monomers are linked by disulfide bonds^14^. In females, AMH is secreted by ovarian granulosa cells^15^ and appears to regulate the early transition from resting primordial follicles to growing follicles^16^.

AMH signals by binding to a specific type-II receptor (AMHR2)^17^,^18^ that heterodimerizes with one of several type-I receptors (ALK2, ALK3 and ALK6), and recruiting Smad proteins that are translocated to the nucleus to regulate target gene expression^19^. AMH is the only known ligand of AMHR2, suggesting that tissues that express this receptor are likely to be targets of AMH. In females, the distribution and function of AMHR2 have not been extensively studied beyond its role in the ovaries, despite its expression in extragonadal tissues including the developing brain^20^,^21^.

Here we show that AMH induces LH secretion by stimulating the activity of the hypothalamic GnRH neurons, which express AMH receptors *in vivo* both in mice and in humans. These data pinpoint AMH as an important regulator of the GnRH system, opening new avenues for the study of AMH signalling in reproductive function and dysfunction.

## Results

### AMHR2 expression in the brain and in GnRH neurons

**Figure 1:**
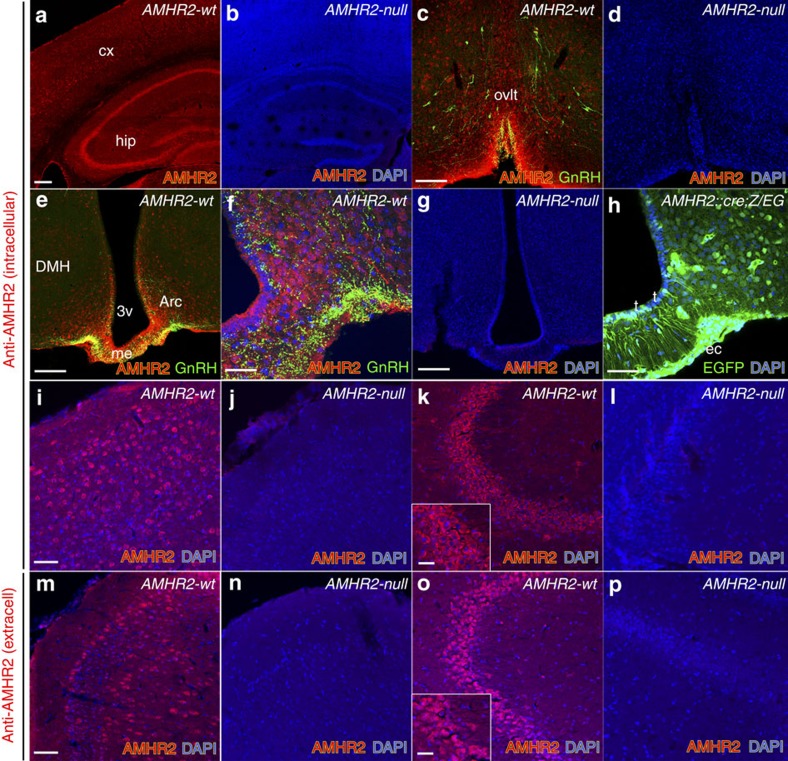
Specificity tests of anti-AMHR2 antibody and identification of AMHR2-expressing cells in the adult mouse brain. (**a**–**p**) Coronal sections immunolabelled with the indicated antibodies, from AMHR2::Cre^+/+^ (AMHR2-wt, number of immunostained P120 female brains, *n*=5) and AMHR2::Cre ^−/−^ (AMHR2-null, number of immunostained P120 female brains, *n*=5). AMHR2 expression was also analysed in AMHR2::Cre^+/*−*^;Z/EG reporter line (**h**; number of immunostained P60 female brains, *n*=3). The vector of Z/EG mouse line was designed to provide lacZ expression before Cre excision and EGFP expression after Cre excision and is referred to as Z/EG (lacZ/EGFP)^23^. AMHR2 expression was found in the cortex (**a**,**i**,**m**), dentate gyrus of the hippocampus (**a**,**k**,**o**), organum vasculosum of the lamina terminalis (OVLT; **c**) hypothalamic median eminence (me) and dorsomedial hypothamaus (DMH) arcuate nucleus (Arc; **e**,**f**,**h**). Moreover, AMHR2-expressing cells were found in hypothalamic tanycytes (t) lining the third ventricle (3V) and endothelial (EC) cells (**f**,**h**). Brains of AMHR2-null mice were totally depleted of AMHR2 immunoreactivity when using an antibody against an intracellular epitope of the AMHR2 (**b**,**d**,**g**,**j**,**l**) or an antibody against the extracellular region of the receptor (**n**,**p**). Experiments were replicated at least three times. Scale bars, (**a**,**b**) 200 μm; (**c**–**e**,**g**) 100 μm; (**f**,**h**) 25 μm; (**i**–**p**) 40 μm (inset in **k** and **o**). DAPI, 4′,6-diamidino-2-phenylindole.

We first examined the expression of AMHR2 protein in the adult brain of *AMHR2* wild-type and null mice (*AMHR2::Cre*)^22^ by labelling the brain sections from postnatal day 90 (P90) female mice with two different antisera to AMHR2: an affinity-purified custom-made rabbit polyclonal antiserum directed against an intracellular epitope (Fig. 1a–l) and a commercial goat polyclonal antiserum directed against an extracellular region of the receptor (Fig. 1m–p).

AMHR2-immunoreactive cells were widely distributed in several brain regions including the cortex (Fig. 1a,i,m), hippocampus (Fig. 1a,k,o) and hypothalamus where GnRH cell bodies and fibres are located (Fig. 1c,e,f). Immunohistochemical analysis in *AMHR2*-null mouse brains revealed the lack of AMHR2 immunoreactivity (Fig. 1b,d,g,j,l,n,p), confirming the specificity of both antibodies.

Moreover, we detected *AMHR2-Cre* activity using the *Z/EG*^23^ mouse reporter line (Fig. 1h) in the same AMHR2-immunopositive territories. Interestingly, AMHR2 expression was detected in two non-neuronal cell types in the ME that regulate neurohormone secretion by interacting closely with GnRH terminals in this region^24^: endothelial cells and specialized hypothalamic glia called tanycytes (Fig. 1f,h).

**Figure 2:**
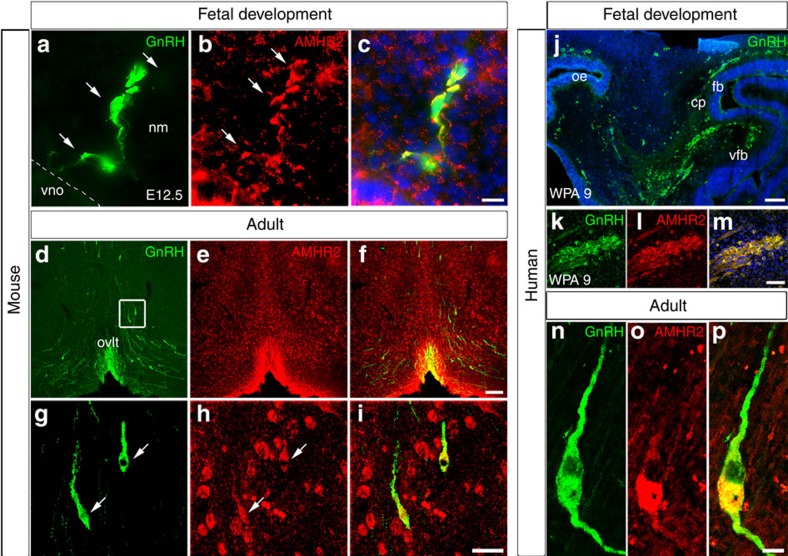
AMHR2 is expressed in mouse and human GnRH neurons. (**a**–**c**) Confocal representative photomicrographs showing GnRH and AMHR2 immunoreactivity in sagittal sections of the E12.5 embryonic nasal compartment (number of immunostained E12.5, *n*=5). Dashed line indicates the boundary between the vomeronasal organ (vno) and the nasal mesenchyme (nm). GnRH neurons migrating out of the vno express AMHR2. (**d**–**i**) Confocal photomicrographs showing GnRH (**d**,**g**) and AMHR2 (**e**,**h**) immunoreactivity in coronal sections through the hypothalamus of adult female mice (P90–120; number of immunostained brains, *n*=5). Images show widespread expression of AMHR2 at the level of the organum vasculosum of the lamina terminalis (OVLT). (**g**–**i**) High-magnifications images of boxed area in **d**. Arrows point to GnRH neurons expressing AMHR2. (**j**–**m**) Representative sagittal section of a human fetus at 9 weeks LMP, immunolabelled for GnRH and AMHR2 (number of immunostained fetuses, *n*=3). At this developmental stage, the majority of GnRH neurons are still located in the nasal region, at the beginning of their migratory path. Double immunofluorescence shows co-expression of these antigens in the same migratory neurons. Experiments were replicated at least three times. cp, cribriform plate; fb, forebrain; oe, olfactory epithelium; vfb, ventral forebrain. (**n**–**p**) Representative coronal section of an adult hypothalamus double labelled for GnRH and AMHR2. Human hypothalami were obtained between 24 and 36 h post mortem from two autopsied individuals: a 20-year-old female and a 72-year-old male subject; 17 out of 29 and 20 out of 35 GnRH neurons exhibited AMHR2 immunoreactivity, respectively (analyses have been performed in eight consecutive 16-μm thick coronal sections for each individual). Scale bars, (**a**–**c**) 10 μm; (**d**–**f**) 100 μm; (**g**–**i**) 20 μm, (**j**) 100 μm, (**k**–**m**) 40 μm, (**n**–**p**) 5 μm.

To determine whether GnRH neurons express AMHR2 during their development and maturation, double-immunofluorescence experiments were performed on the sections of mouse (Fig. 2a–c) and human fetuses (Fig. 2j–m), as well as on the hypothalamic sections from adult female mice (Fig. 2d–i) and humans (Fig. 2n–p). We found that in both species, AMHR2 was expressed in GnRH neurons in early embryonic development as well as in adulthood. In adult mice (Fig. 2d–i) and humans (Fig. 2n–p), double immunolabelling revealed that AMHR2 continued to be expressed by >50% of the GnRH neurons located in the sections containing the preoptic region (POA), including the organum vasculosum of the lamina terminalis, as well as in more rostral areas such as in the septum and in the diagonal band of Broca (Supplementary Fig. 1).

**Figure 3:**
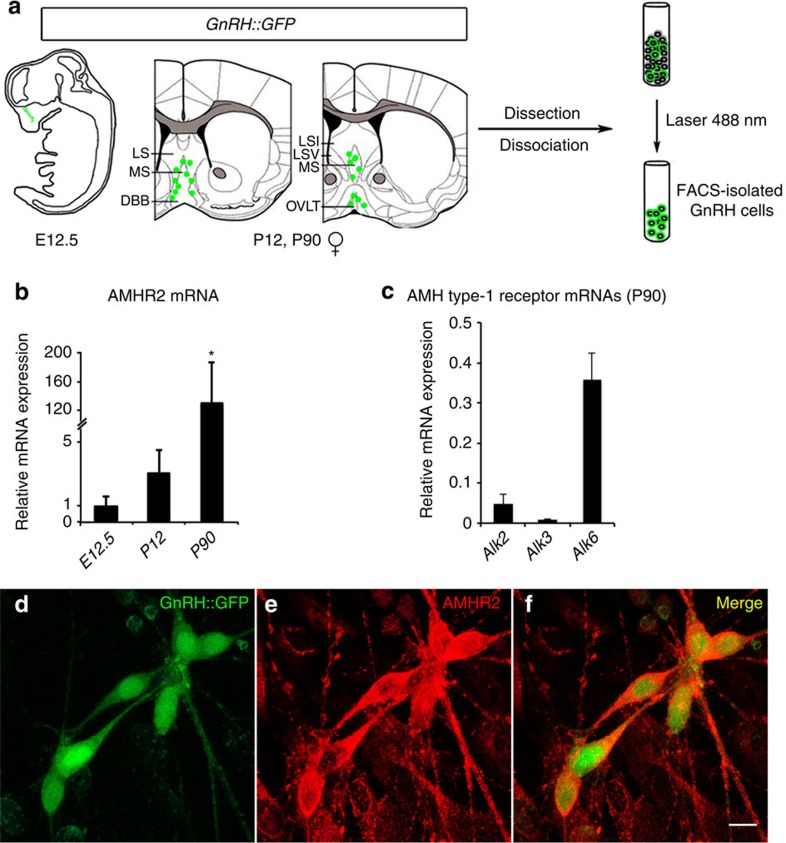
AMH receptor transcript expression in GnRH neurons. (**a**) Schematic summarizing the isolation of GnRH-GFP cells. GFP-positive GnRH neurons were isolated by fluorescence-activated cell sorting from the nasal region of E12.5 embryos (*n*=3) and from the hypothalamic preoptic area of postnatal (females P12, *n*=3) and adult (females P90, *n*=3) female mice. (**b**) Real-time PCR analysis of expression levels of *AMHR2* messenger RNA (mRNA) in GnRH cells sorted at E12.5, P12 and P90. *AMHR2* transcript expression was detectable in GnRH neurons at all stages, although it reached its highest level at P90. Values are expressed relative to values at E12.5, set at 1, and shown as means±s.e.m. One-way analysis of variance, F_(2,8)_=7.6, *P*=0.02. Fisher's least significant difference *post hoc* test, *: *P*<0.05 between P90–P120 and E12.5 and P90–P120 versus P12. (**c**) Relative mRNA expression of AMH type-I receptors (*Alk2*, *Alk3* and *Alk6*) in GnRH neurons isolated from the preoptic region of adult female mice (females P90, *n*=3). All receptor transcripts were detectable in adult GnRH neurons. (**d**–**f**) Nasal explants were generated from E11.5 *GnRH-GFP* embryos (*n*=7) and maintained in serum-free media for 7 days before immunohistochemistry procedures. Immunostaining for AMHR2 show co-localization with fully differentiated GnRH-GFP neurons. Experiments were replicated three times. LS, lateral septum; MS, medial septum; DBB, diagonal band of Broca; LSI, lateral septal nucleus, intermediate part; LSV, lateral septal nucleus, ventral part; OVLT, organum vasculosum of the lamina terminalis. Scale bars, (**d**–**f**) 10 μm.

We next took advantage of *GnRH::GFP*^25^ mice to isolate green fluorescent protein (GFP)-positive GnRH neurons at different developmental stages by fluorescence-activated cell sorting (FACS)^26^ (Fig. 3a) and used quantitative reverse transcription–PCR (RT–PCR) to analyse the expression of genes of interest in these neurons. At embryonic day 12.5 (E12.5), when most GnRH neurons are still located in the nasal region, where they are born^27^,^28^, *AMHR2* expression was low. However, in the hypothalamic/preoptic area of juvenile (postnatal day 12; P12) and adult female mice (P90), in which GnRH neurons have completed their migration into the brain, *AMHR2* transcript levels were significantly increased (Fig. 3b). In addition, mature GnRH neurons expressed the three ALK receptors (Fig. 3c).

We then confirmed by immunocytochemistry the AMHR2 protein expression in fully differentiated primary *GnRH::GFP* neurons^29^,^30^, using an antibody directed against the extracellular region of the receptor (Fig. 3d–f).

### AMH regulates GnRH neuron excitability and secretion

To determine whether AMH acts predominantly at GnRH cell bodies or terminals or both, we investigated the effect of AMH on GnRH neuron firing activity and GnRH secretion in brain slices containing GnRH somata or terminals, respectively. We first performed electrophysiological recordings of GnRH neurons in acute brain-slice preparations containing the POA of *GnRH::GFP* mice. AMH at 0.04, 0.4 and 4 nM (equivalent to 1, 10 and 100 ng ml^−1^ of recombinant AMH, homodimeric active form) concentrations was tested on GnRH-GFP neuronal cell bodies located in the POA by bath application. These concentrations are in the physiological range of serum AMH that was previously reported to be ∼0.1 nM (for the 140-kDa homodimeric precursor) in 4–8-month-old female mice^31^.

**Figure 4:**
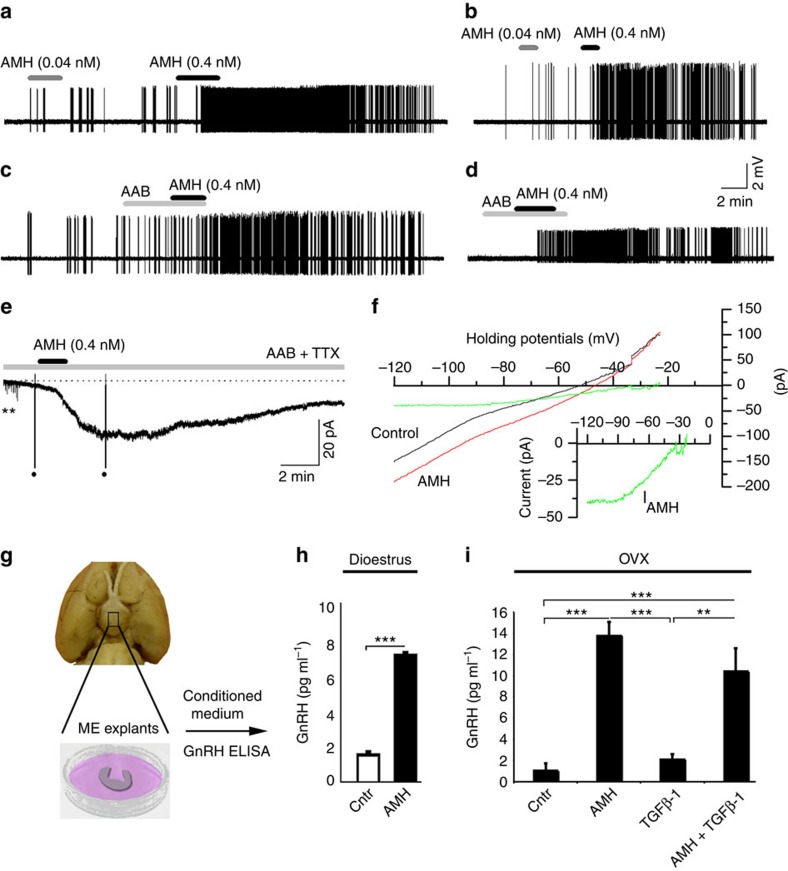
AMH increases GnRH neuron firing and hormone secretion. (**a**,**b**) Cell-attached voltage recordings of GnRH neurons from *GnRH::GFP* male mice stimulated with AMH. (**c**,**d**) Cell-attached voltage recordings of GnRH neurons from *GnRH::GFP* diestrous mice. AAB (AP5, CNQX and GABAzin) application shows that AMH excitation of GnRH neurons is not dependent upon amino-acid transmission. (**e**) Whole-cell current recording of a GnRH neuron in the presence of AAB and tetrodotoxin (TTX). **Spontaneous synaptic currents blocked by AAB+TTX. Dots indicate truncated currents induced by the ramp voltage protocol. (**f**) Current–voltage plots before (black) and during AMH response (AMH, red). The ‘AMH current' (I_AMH_, green) is obtained by the subtraction of control from AMH current at ramp potentials (−120 to −20 mV for 1.5 s). AMH current with a reversal potential around −30 mV is replotted as an inset with an expanded current axis. (**g**) Schematics illustrating ME dissection and explant preparation. Hypothalamic MEs were microdissected from adult diestrous or ovariectomized (OVX) female rats. (**h**) Quantification of GnRH secretion from ME explants of P120 diestrous rats stimulated or not with 125 nM AMH (Die, *n*=4; Die+AMH, *n*=4). GnRH mean concentration±s.e.m. Unpaired Student's *t*-test, *t*_(6)_=−6.01, ***P<0.001. (**i**) MEs dissected from OVX rats (*n*=4 each group). One-way analysis of variance, F_(3,15)_=30.9, *P*<0.0001. ***P<0.0001, **P<0.001 Fisher's least significant difference *post hoc* test. All the experiments were replicated at least three times.

At 0.04 nM, 8 out of 20 GnRH neurons (2 male, 3 dioestrus) exhibited a mean increase in firing rate from 0.82±0.36 to 1.76±0.38 Hz (values are mean±s.e.m.; *P*=0.0142; Wilcoxon signed-ranks test for paired samples) that lasted for 6.12±1.67 min (Fig. 4a,b). The eight responding cells were then tested with 0.4 nM AMH, which induced a more marked increase in neuronal activity (firing rate: 1.25±0.43 to 2.75±0.37 Hz, *P*=0.0142; Wilcoxon signed-ranks test) with a mean duration of 12.7±1.4 min (Fig. 4a,b). Another group of 10 GnRH neurons (2 proestrus and 2 dioestrus) was tested with 4 nM AMH with 4 of 10 displaying an increase in excitation from 0.93±0.55 to 2.47±0.24 Hz (*P*<0.05, Wilcoxon signed-ranks test for paired samples), with the duration of enhanced activity lasting for 13.2±1.2 min.

To determine whether the effects of AMH on GnRH neurons required ionotropic amino-acid transmission, an amino-acid receptor blocker (AAB) cocktail consisting of the AMPA/kainate antagonist CNQX (20 μM), the NMDA and AMPA/kainate receptor antagonist kynurenic acid (2 mM) and the GABA_A_ receptor antagonist GABAzine (5 μM) was applied to the bath before AMH application. AMH (0.4 nM) continued to increase GnRH neuronal firing in the presence of the cocktail (*n*=10; 2 dioestrus and 4 proestrus; Fig. 4c,d). To determine whether the effects of AMH were direct on GnRH neurons, whole-cell recordings of 19 GnRH neurons (4 male and 2 dioestrus mice) were undertaken in the AAB cocktail with the addition of 0.5 μM TTX. AMH at 0.4 nM continued to evoke an inward current in six GnRH neurons (Fig. 4e) with a peak amplitude (holding at −60 mV) of 20±6 pA (range from 9 to 47 pA) accompanied by an increase in conductance and a reversal potential of −32±4 mV (Fig. 4f).

We next tested GnRH neurons from 4 male (*n*=9 cells), 5 diestrous female (*n*=11 cells) and 6 proestrous female (*n*=24 cells) mice with the sub-maximal concentration of AMH (0.4 nM) to determine whether the response of GnRH neurons to AMH differed according to sex or oestrous cycle phase. In all groups, ∼50% of GnRH neurons responded to AMH (56% in males, 64% in diestrous and 49% in proestrous females) with a 70–80% increase in activity that lasted for 10–17 min (Table 1). The percentage of cells responding and the magnitude of the increase in firing were not different between the three groups (*χ*^2^-test and analysis of variance). However, the duration of the response was significantly reduced in GnRH neurons from proestrous mice compared with diestrous animals (*P*<0.01; Fisher's Least Significant Difference; Table 1).

Together, these electrophysiological investigations show that AMH exerts a powerful stimulatory influence on GnRH neuron excitability. Actions of AMH on approximately half of all GnRH neurons in male and female mice are direct and potent occurring in the subnanomolar range.

We next studied whether AMH could also act at the level of GnRH nerve terminals in the ME, to modulate hormone secretion (Fig. 4g,h). ME explants, which contain only GnRH neuron axon terminals, but not cell bodies, were generated as previously described^32^,^33^ from adult female rats during dioestrus, when GnRH secretion is low, and challenged them with AMH for 4 h before the use of enzyme-linked immunosorbent assay (ELISA) to measure the amount of GnRH secreted into the medium (Fig. 4h).

In explants from diestrous rats, treatment with AMH resulted in a fourfold increase in GnRH release when compared with vehicle-treated explants (Fig. 4h). These results were confirmed by repeating the same experiments in adult female rats 4 weeks after they were ovariectomized, to determine whether AMH had the same effect in the absence of hormones secreted by the ovaries (Fig. 4i).

Since AMH is a member of the TGF-β superfamily, we further tested whether a related peptide TGF-β1 could elicit a similar increase in GnRH secretion from the ME of ovariectomized rats (Fig. 4i). In agreement with earlier studies^33^, TGF-β1 treatment did not alter GnRH release (Fig. 4i), confirming the specificity of the AMH signalling pathway in the increase in GnRH neuronal activity and hormone secretion.

### Central AMH administration stimulates LH secretion *in vivo*

**Figure 5:**
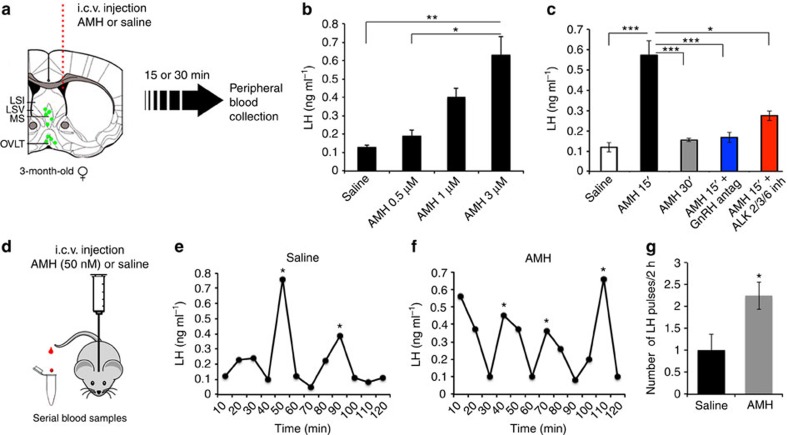
Intracerebroventricular (i.c.v.) administration of AMH *in vivo* increases the LH secretion. (**a**) Schematic depicting AMH injection into the lateral cerebral ventricle of diestrous mice. Trunk blood was collected from 3–4-month-old mice 15 or 30 min after the injection. (**b**) Following AMH administration (AMH 0.5 μM, *n*=3; AMH 1 μM, *n*=4; AMH 3 μM, *n*=6; saline, *n*=5), LH was measured. Values are expressed as means±s.e.m. One-way analysis of variance, F _(3,19)_=6.6, *P*=0.004. *P:<0.01, **P:<0.001, Fisher's least significant difference *post hoc* test. (**c**) Adult diestrous mice were injected with AMH 3 μM (*n*=20), or saline (*n*=7). Plasma LH secretion peaked at 15 min after injection and returned to basal levels 30 min later (*n*=7). AMH was injected i.c.v 2 h after an intraperitoneal injection of the GnRH antagonist (*n*=7). Treatment with an ALK receptor inhibitor prevented the AMH-dependent increase in LH secretion (*n*=6). One-way analysis of variance, F_(4,47)_=11.1, *P*<0.0001. *P<0.01, ***P<0.0001, Fisher's least significant difference *post hoc* test. (**d**) Schematic representation of AMH injection into the lateral cerebral ventricle of diestrous (3–4-month old) mice with 50 nM AMH or saline. Tail blood was collected every 10 min for 2 h and LH measured (*n*=7 control, *n*=8 AMH; **e**–**g**). Asterisks in **e** and **f** indicate LH pulses in two -representative saline- and AMH-treated mice. Values are represented as means±s.e.m. Unpaired Student's *t*-test, *t*_(13)_=−2.56, *P<0.05. Experiments were replicated three times. MS, medial septum; LSI, lateral septal nucleus, intermediate part; LSV, lateral septal nucleus, ventral part; OVLT, organum vasculosum of the lamina terminalis.

To examine the effects of AMH on gonadotropin secretion *in vivo*, we administered AMH directly into the lateral ventricle of diestrous female mice, and measured LH secretion, which is an index of GnRH release (Fig. 5a). We first analysed plasma LH concentrations 15 min after administering increasing doses of AMH (Fig. 5b), and found that 3 μM of recombinant AMH injected intracerebroventricularly (i.c.v.) induced the strongest increase in LH release 15 min after the injection.

We thus used this concentration in subsequent experiments, and analysed LH levels 15 and 30 min after the injection. AMH administration induced a rapid increase in LH secretion 15 min after treatment (Fig. 5c), which returned to baseline by 30 min, suggesting that this AMH effect is not mediated by the canonical Smad proteins, whose activation normally requires a few hours, but rather through a fast and non-genomic pathway. Interestingly, the effects of AMH treatment on LH secretion were significantly attenuated by the intravenous delivery of an ALK 2/3/6 inhibitor (100 μM) 2 h before, indicating that the AMH-induced rise in LH requires AMH receptor signalling (Fig. 5c).

To determine whether the actions of AMH administered i.c.v. on GnRH/LH secretion were indeed mediated by GnRH neuronal activity and not by its transcellular transport to the pituitary portal circulation, thus bypassing GnRH neurons, we administered a GnRH antagonist (cetrorelix acetate; 0.5 mg Kg^−1^), intraperitoneally (i.p.) 30 min before AMH i.c.v. administration. Cetrorelix acetate is known to specifically saturate GnRH receptors at the level of the anterior pituitary^34^,^35^, thus preventing GnRH-mediated LH secretion. The effects of AMH on LH secretion were completely blocked by cetrorelix treatment, excluding a direct effect of AMH at the level of the pituitary, where both the ligand and its receptor are expressed and active in gonadotropin transcription^36^. This provides further support for the central action of AMH on GnRH neurons (Fig. 5c).

The lowest concentrations of AMH tested were ineffective in inducing an acute LH rise 15 min following the i.c.v. injection (Fig. 5b). We thus tested whether low doses of recombinant AMH (50 nM) were able to affect the speed of the GnRH/LH pulse generator, a neuroendocrine feature of PCOS. Using a sensitive ELISA to measure LH in 5 μl of tail blood^37^, we measured LH pulses, identified by peak values >2 s.d.'s above baseline, in serial blood samples collected every 10 min after AMH i.c.v. injection (Fig. 5d). Compared with dioestrus controls (*n*=7; Fig. 5e,g), AMH significantly increased the number of LH peaks/2 h (*n*=8; Student's *t*-test *P*<0.05; Fig. 5f,g).

### AMH and LH levels correlate in a PCOS mouse model

Using a prenatal androgen (PNA)-treated mouse model of PCOS^38^,^39^,^40^, which similarly to the clinical syndrome, displays increased LH pulse frequency, as well as impaired oestrous cyclicity^40^, we next aimed to analyse (1) whether AMH levels are increased in the PNA female mice and (2) whether a positive or negative correlation exists between changes in AMH, LH and FSH in these mice.

Hormonal levels were assessed in control (*n*=10; 3–4-month old) and PNA animals (*n*=8; 3–6-month old) during dioestrus. Unlike the human syndrome, the mean AMH plasma concentration did not differ between the two groups (Ctrl: 19.83 ng ml^−1^±3.37; PNA: 19.9 ng ml^−1^±3.33; Student's *t*-test: *P*=0.6). However, we found that AMH and LH levels were significantly and positively correlated in the PNA-treated mice (Pearson *R*^2^=0.71; *P*<0.01) but not in the Ctrl animals (Pearson *R*^2^=8.4E−06; *P*=0.9).

The mean FSH plasma concentration did not differ between the two groups (Ctrl: 0.37 ng ml^−1^±0.01; PNA: 0.36 ng ml^−1^±0.06; Student's *t*-test: *P*=0.8) and, in addition to that, we did not observe any correlation between AMH and FSH levels in any experimental conditions (Ctrl *n*=10; Pearson *R*^2^=0.033; *P*=0.6; PNA *n*=8; Pearson *R*^2^=0.174; *P*=0.3).

## Discussion

It is known that dysregulation of gonadotropin secretion is associated with various human fertility disorders, among which is the common reproductive disorder PCOS. PCOS patients indeed show high LH pulse frequency and low FSH secretion^6^, although the origin of this dysregulation is unknown. Here we demonstrate that AMH, whose circulating levels are abnormally elevated in the majority of the PCOS patients, increases GnRH neuronal activation and neurohormone secretion.

The expression of the AMHR2 has already been described outside of the gonads, including in the endometrium^41^, breast^42^, prostate^43^ and cervix^44^ in humans, and in the developing brain^21^ and fetal lungs^45^ in mice. Our neuroanatomical data expand these results by showing that AMHR2 is expressed in several adult brain regions including the hippocampus, cortex and hypothalamus.

Although AMH signalling has been reported to play a crucial role during sex differentiation and gonadal functions^46^,^47^, accumulating evidence has started to shed light on unexpected functions of AMH in neural network formation, spanning from neuroprotective roles in cortical and motor neurons to the initiation of sex-linked bias and sexually dimorphic hypothalamic areas^20^,^21^,^48^,^49^,^50^.

These studies suggest that AMH could be a regulator of neural network formation, however, the definition of its actions will require detailed investigations of many different types of neurons during development and in postnatal life.

We demonstrate here that AMHR2 is expressed in a significant subset of hypothalamic GnRH neurons both in mice and in humans and that AMH directly activates the firing activity of approximately half of this cellular population located in the POA in mice. Interestingly, these actions of AMH were shown to be effective at subnanomolar concentrations (from 0.04 to 4 nM), evoking direct and robust electrophysiological responses in GnRH neurons, that depended neither on the stage of the oestrous cycle nor on the sex.

Our experimental data also show that AMH activates directly 50–64% of GnRH neurons in a dose-related manner. Notably, only 50–70% of all GnRH neurons are thought to be involved in controlling pituitary gonadotropin secretion^51^,^52^, indicating that AMH might indeed be relevant in the regulation of GnRH/LH secretion. This is particularly relevant since previous work demonstrated that only 12% of the GnRH neuron population is required for pulsatile gonadotropin secretion and puberty onset, whereas between 12 and 34% are required for cyclical control in adult female mice^53^.

To examine the effects of AMH on gonadotropin secretion *in vivo*, we administered AMH directly into the lateral cerebral ventricle of the brain of female mice and demonstrated that it increases GnRH-dependent LH pulsatility and secretion, providing further physiological support for our electrophysiological and *in vitro* evidence and strengthening the notion of a central action of AMH on GnRH neurons. The apparent dissociation between the *in vitro* potency of AMH and the *in vivo* responses, which are engaged by higher doses of AMH, are not surprising as they most likely reflect the different accessibility of the recombinant protein to the receptors in living brain slices *in vitro* when compared with *in vivo* experiments.

AMH is secreted as a 140-kDa homodimeric precursor (70-kDa monomers). Each monomer consists of two parts: a 25-kDa COOH-terminal dimer, which becomes bioactive after proteolytic cleavage and binds the AMHRII^54^,^55^, and a pro-region, which is important in AMH synthesis and the extracellular transport. Notably, specialized fenestrations of the endothelial cells in the ME allow the diffusion of molecules below 40 kDa (refs 56, 57). Thus, bioactive AMH could access this region through the fenestrated capillaries and act either directly on GnRH nerve terminals allowing rapid modulation of GnRH release and/or indirectly via AMHR2-expressing tanycytes or endothelial cells. The first hypothesis is partly supported by our *in vitro* findings showing that AMH induces GnRH secretion from ME explants, even in the absence of the GnRH cell bodies. In addition to that, GnRH neurons extend dendritic trees beyond the blood–brain barrier into the organum vasculosum of the lamina terminalis^58^, where they could also directly sense molecules circulating in the bloodstream^59^.

Using a preclinical murine model of PCOS^38^,^39^,^40^, we also found a positive correlation between AMH and LH levels, even though we did not detect any significant elevation of the mean serum AMH in the PNA mice. These data are in agreement with previous clinical studies in which a positive correlation was found in AMH and LH, but not between AMH and FSH response dose, in patients with PCOS or with normogonadotropic anovulatory infertility^60^,^61^. In recent work, Kissell *et al.*^62^ documented a strong association between AMH levels and sporadic anovulation in a large cohort of healthy women. Similarly to the previous work demonstrating higher serum AMH concentrations from women with known anovulatory PCOS^13^, these authors described women with sporadic anovulatory cycles to have elevated AMH levels compared with ovulatory women.

**Figure 6:**
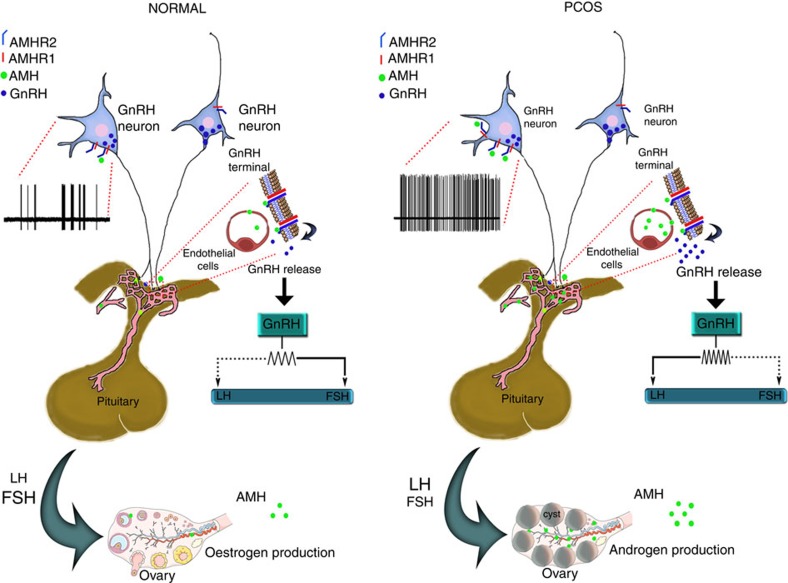
Schematic representation of the proposed mechanism of action of AMH on GnRH neurons in normal and PCOS women. In normal women of reproductive age, the levels of circulating AMH are low and do not significantly fluctuate over the menstrual cycle. GnRH neurons express AMHR2 as well as type-I AMH receptors (ALK 2/3/6). In women with PCOS, circulating AMH levels are two to three times higher than in normal women. We hypothesize that AMH could bypass the blood–brain barrier by passing through the fenestrated capillaries at the level of the organum vasculosum of the lamina terminalis (OVLT) and the median eminence and act directly on GnRH dendrites and terminals, respectively. In the median eminence, AMH could also act indirectly on GnRH neurons via tanycytes and vascular endothelial cells, which also express AMHR2, possibly contributing with other dysregulated factors to the increase in GnRH and LH pulsatilities. The altered ratio of LH to FSH is known to be responsible for the ovarian androgen production, partly explaining the two central diagnostic features of PCOS: hyperandrogenemia and hyperandrogenism (hirsutism). FSH, follicle-stimulating hormone; LH, luteinizing hormone.

Here we put forward the appealing hypothesis that elevated AMH plasma levels, which characterize many PCOS patients, might contribute to the hormonal alterations observed in PCOS, such as a significant rise in LH secretion (Fig. 6). Elevated LH pulsatility is known to be responsible for increased ovarian androgen production by theca cells, which could then partly explain the two central diagnostic features of PCOS: hyperandrogenemia and hyperandrogenism (hirsutism). In addition, in PCOS, ovarian hyperandrogenism, hyperinsulinemia due to insulin resistance, and altered intraovarian AMH signalling severely impact dominant follicle selection and follicular growth. The consequent follicular arrest in PCOS is accompanied by menstrual irregularities, sporadic ovulation or anovulation and the accumulation of small antral follicles within the periphery of the ovary, leading to the two other diagnostic features of PCOS: oligoanovulation and ovaries with a polycystic morphology^63^ (Fig. 6). Although this working hypothesis is attractive, future studies are required to determine the eventual pathophysiological role of AMH in the neuroendocrine dysregulation underlying PCOS. Indeed, we do not currently know whether elevated AMH is the driving force behind all the different series of hormonal changes in PCOS, including the elevated LH:FSH ratio. Nevertheless, previous clinical studies have revealed normal pituitary gonadotropin profiles, but significantly elevated serum AMH concentrations, in prepubertal daughters of women with PCOS^64^,^65^. It is known that a significant proportion of PCOS daughters develop early reproductive and metabolic disturbances as endocrine antecedents to adult PCOS^63^. These observations thus suggest that AMH levels might increase before the rise in LH in women at risk of developing PCOS. Prospective epidemiological studies on a large cohort of subjects are needed to establish whether the postmenarcheal girls with elevated AMH levels ultimately develop the syndrome.

This study establishes a new role for the AMH as a central regulator of the hypothalamic–pituitary–gonadal axis under both physiological and pathological conditions, and raises the intriguing hypothesis that the perturbation of the AMH-dependent regulation of GnRH release could play a critical role in the development of PCOS.

## Methods

### Animals

Female Sprague Dawley rats and C57BL/6J mice (Charles River, USA) were housed under specific pathogen-free conditions in a temperature-controlled room (21–22 °C) with a 12-h light/dark cycle and *ad libitum* access to food and water. *AMHR2-Cre* and *Z/EG*^*loxP−βgeo/+*^ (LacZ/EGFP) knock-in mice have been previously characterized^22^,^23^. *GnRH::GFP*^25^ mice were a generous gift of Dr Daniel J Spergel (Section of Endocrinology, Department of Medicine, University of Chicago, IL). Animal studies were approved by the Institutional Ethics Committees of Care and Use of Experimental Animals of the Universities of Lille 2 (France) and the University of Otago School of Medical Sciences, Dunedin (New Zealand). All experiments were performed in accordance with the guidelines for animal use specified by the European Union Council Directive of 22 September 2010 (2010/63/EU) and with the regulations of Australian and New Zealand Council for the Care of Animals in Research and Teaching.

### Human tissues

Human fetuses (9 gestation weeks, *n*=3) were obtained from voluntarily terminated pregnancies, with the parent's written informed consent. Tissues were made available in accordance with French bylaw (good practice concerning the conservation, transformation and transportation of human tissue to be used therapeutically, published on 29 December 1998). Permission to utilize human brain tissues was obtained from the French agency for biomedical research (Agence de le Biomédecine, Saint-Denis la Plaine, France). The fetuses were immersion fixed in 4% paraformaldehyde (PFA) in 0.1 M PB (pH 7.4) for 3 weeks, cryoprotected in 30% sucrose in PBS for 48 h, embedded in Tissue-Tek (Miles, Elkhart, IN) and frozen in liquid nitrogen. Adult human hypothalami were obtained between 24 and 36 h post mortem from two autopsied individuals: a 20-year-old female subject and a 72-year-old male subject, who had both donated their bodies to science in accordance with French bioethics laws. A review of their medical records indicated that they had no known neurological or neuroendocrinological disorders, and the cause of death was cardiac and respiratory failure, respectively. The hypothalami were isolated and immersion fixed in 4% PFA in 0.1 M PB (pH 7.4) for 1 week, cryoprotected in 20% sucrose in PBS for 48 h, embedded in Tissue-Tek (Miles, Elkhart, IN) and frozen in liquid nitrogen.

### Tissue preparation

For immunohistochemical analysis, embryos (embryonic day 12.5) were obtained after cervical dislocation from timed-pregnant C57BL/6J mice. Embryos were washed thoroughly in cold 0.1 M PBS, fixed in fixative solution (4% PFA, 0.2% picric acid in 0.1 M PBS, pH 7.4) for 4 h at 4 °C and cryoprotected in 30% sucrose overnight at 4 °C. The following day, embryos were embedded in OCT-embedding medium (Tissue-Tek), frozen on dry ice and stored at −80 °C until sectioning. Adult female mice and rats (3–4-month old) were anaesthetized with 100 mg kg^−1^ of ketamine-HCl and 10 mg kg^−1^ xylazine-HCl and perfused transcardially with 20 ml of saline, followed by 100 ml of 4% PFA, pH 7.4. Brains were collected, post-fixed in the same fixative for 2 h at 4 °C, embedded in OCT-embedding medium (Tissue-Tek), frozen on dry ice and stored at −80 °C until cryosectioning.

### Immunohistochemistry

Tissues were cryosectioned (Leica cryostat) at 16 μm except for mouse adult brain tissues, which were cut at 35 μm for free-floating sections. Tissue sections or cultures were blocked in PBS with 5% normal donkey serum (D9663; Sigma) and 0.3% Triton X-100 (Sigma) for 1 h at room temperature before incubation with different primary antibodies (mentioned in antibodies index below) and then washed extensively in PBS and exposed to corresponding secondary antibodies, Alexa-Fluor 488-conjugated (1:400; Life Technologies, reference numbers: R37118, A-11055, A-11073) and Cy3-conjugated (1:800, Jackson Immuno Research Laboratories, reference numbers: 711-165-152, 705-165-147, 706-165-148) secondary antibodies (Invitrogen), for 1 h in 5% normal donkey serum. After washes, the samples were incubated for 2 min with 0.02% Hoechst 33258 (H3569; Invitrogen) in PBS for fluorescent nuclear staining, and mounted on glass slides and coverslipped with Permafluor medium (Reference number: 434990; Immunon, Pittsburgh, PA). Control sections were incubated in the absence of a primary antibody and on brain sections of deficient mice (*AMHR2* knockout animals). The primary antisera used were as follows: rabbit anti-GnRH (1:3000), a generous gift from Professor G Tramu (Centre Nationale de la Recherche Scientifique, URA 339, Université Bordeaux I, Talence, France)^66^, guinea-pig anti-GnRH (1:10000), a generous gift from Dr Erik Hrabovszky (Laboratory of Endocrine Neurobiology, Institute of Experimental Medicine of the Hungarian Academy of Sciences, Budapest, Hungary), chicken anti-vimentin (1:2000, AB5733, Millipore), polyclonal rabbit anti-AMHR2 (1:2,000, custom-made immunogenic peptide: CELWALAVEERKRPNIPS-NH2, intracellular serine/threonine kinase domain, CASLO, Denmark), goat polyclonal rat anti-AMHR2 (1:500, extracellular domain of the receptor, AF 1618R&D systems). Primary antibodies were diluted in 0.01 M PBS, pH 7.4, 0.5% TritonX-100, and 1% normal serum, made in the same host species of the secondary antibody. For AMHR2, microwave epitope retrieval treatment in citrate buffer, pH ∼6, was performed before immunolabelling of mouse brain sections.

Sections were examined using an Axio Imager.Z1 ApoTome microscope (Carl Zeiss, Germany) equipped with a motorized stage and an AxioCam MRm camera (Zeiss). For confocal observation and analyses, an inverted laser scanning Axio observer microscope (LSM 710, Zeiss) with an EC Plan NeoFluor × 100/1.4 numerical aperture oil-immersion objective (Zeiss) was used (Imaging Core Facility of IFR114, of the University of Lille 2, France).

### Determination of GnRH secretion

Adult female rats (4-month old) with regular 4-day oestrous cycle were killed on the day of dioestrus and ME explants were dissected and processed as follows: ME explants were incubated in artificial cerebrospinal fluid (aCSF) with the following composition (in mM): NaCl, 117; KCl, 4.7; NaH_2_PO_4_, 1.2; NaHCO_3_, 25; CaCl_2_, 2.5; MgCl_2_, 1.2; glucose, 10, bubbled with 95% O_2_-5% CO_2_. (pH 7.4, osmolarity 304 mOsm). Medium was collected before and after explants were treated with recombinant human anti-Müllerian Hormone (AMH, R&D Systems, 125 nM), recombinant human TGFβ-1 (Millipore, 2 nM) or both, followed by incubation at 37 °C for 4 h at 300 r.p.m. The same concentration of TGFβ-1 has previously been used to evaluate GnRH secretion from ME explants^33^. Explants were treated with 0.05 M KCl at the end of the incubation period to confirm their viability. Collected media were analysed for GnRH content following a GnRH ELISA protocol (Phoenix Pharmaceuticals Inc., California, Catalog no. # FEK-040-02).

### Intracerebroventricular injections

The mice were placed in a stereotactic frame (Kopf Instruments, California) under anaesthesia (isoflurane), and a burr hole was drilled 1.7 mm posterior to the bregma, according to a mouse brain atlas. A 10-μl Hamilton syringe was slowly inserted into the lateral ventricle (5.6 mm deep relative to the dura), and 1.5 μl of saline or human recombinant AMH (R&D Systems, 50 nM, 0.5, 1 or 3 μM per mouse) was injected using a perfusion pump over 5 min.

### Rodent LH and FSH and AMH ELISA

Plasma LH was measured using a sensitive sandwich ELISA^37^ with a theoretical detection range of whole blood mLH (in a 1:30 dilution) of 0.117–30 ng ml^−1^. The intra- and interassay coefficients of variation were 6.05% and 4.29%, respectively. The GnRH antagonist cetrorelix acetate (Sigma, 0.5 mg Kg^−1^) was injected i.p. 1 h before i.c.v. injection, while the ALK inhibitor (dorsomorphin dihydrochloride, Tocris, 100 μM) was injected intravenously 2 h before. Animals were killed by cervical dislocation and trunk blood was collected in sterile Eppendorf tubes and left on ice until centrifugation, plasma was frozen and stored at −80 °C until use. Plasma FSH and AMH levels were measured using, respectively, a commercial ELISA kit (Endocrine Technologies, Inc.) and a competitive ELISA kit (CUSABIO; #CSB-E13156m), following manufacturer's instructions.

### Fluorescence-activated cell sorting and analysis

Embryos were collected at E12.5 from timed-pregnant *GnRH::GFP* mice anaesthetized with an i.p. injection of 100 mg kg^−1^ of ketamine-HCl and killed by cervical dislocation. Juvenile (P12) and adult female mice (P90) were anaesthetized with 50–100 mg kg^−1^ of ketamine-HCl and 5–10 mg kg^−1^ xylazine-HCl before being killed by cervical dislocation. Microdissected tissue from the embryonic nasal region and the hypothalamic POA of juvenile/adult animals were enzymatically dissociated using the Papain Dissociation System (Worthington, Lakewood, NJ) to obtain single-cell suspensions following manufacturer's instructions. After dissociation, the cells were physically purified using a FACSAria III (Beckman Coulter) flow cytometer equipped with FACSDiva software (BD Biosciences). The sort decision was based on measurements of GFP fluorescence (excitation: 488 nm, 50 mW; detection: GFP bandpass 530/30 nm, autofluorescence bandpass 695/40 nm) by comparing cell suspensions from *GnRH::GFP* and wild-type animals. For each animal, 500 GFP-positive cells were sorted directly into 8 μl of extraction buffer: 0.1% Triton X-100 (Sigma-Aldrich) and 0.4 U μl^−1^ RNaseOUT (Life Technologies). Captured cells were used to synthesize first-strand complementary DNA using the SuperScript III First-Strand Synthesis System for RT–PCR (Invitrogen) following the manufacturer's instructions. Controls without reverse transcriptase were performed to demonstrate the absence of contaminating genomic DNA.

### Quantitative RT–PCR analyses

For gene expression analyses, messenger RNAs obtained from FACS-sorted GnRH neurons were reverse transcribed using SuperScript III Reverse Transcriptase (Life Technologies) and a linear preamplification step was performed using the TaqMan PreAmp Master Mix Kit protocol (Applied Biosystems). Real-time PCR was carried out on Applied Biosystems 7900HT Fast Real-Time PCR System using exon-boundary-specific TaqMan Gene Expression Assays (Applied Biosystems): *Gnrh1* (Gnrh1-Mm01315605_m1), *Amhr2* (AMH2r-Mm00513847_m1), *Alk2* (Acvr1-Mm01331069_m1), *Alk3* (Bmpr1a-Mm00477650_m1), *Alk6* (Bmpr1b-Mm03023971_m1), *Smad 1* (Smad1-Mm00484723_m1), *Smad 4* (Smad4-Mm03023996_m1), *Smad 5* (Smad5- Mm03024001_g1) and *Smad 8* (Smad8/9-Mm00649885_m1). Control housekeeping genes: *r18S* (18S-Hs99999901_s1); *Actb* (Actb-Mm00607939_s1). Quantitative real-time PCR was performed using TaqMan Low-Density Arrays (Applied BioSystems) on an Applied BioSystems 7900HT thermocycler using the manufacturer's recommended cycling conditions. Gene expression data were analysed using SDS 2.4.1 and Data Assist 3.0.1 software (Applied BioSystems).

### Primary GnRH cultures (nasal explants)

Nasal pits of E11.5 *GnRH-GFP* mice were isolated under aseptic conditions in Gey's Balanced Salt Solution (Invitrogen) enriched with glucose (Sigma-Aldrich). Nasal explants were placed onto glass cover slips coated with 10 ml of chicken plasma (Cocalico Biologicals, Inc.). Thrombin (10 ml; Sigma-Aldrich) was then added to allow the explant to adhere to the cover slip (thrombin/plasma clot). Explants were maintained in defined serum-free medium containing 2.5 mg ml^−1^ fungizone (Sigma-Aldrich) at 37 °C with 5% CO_2_ for up to 30 days *in vitro*. From culture day 3 to day 6, fresh media containing fluorodeoxyuridine (8 × 10^*−*5^ M; Sigma-Aldrich) was provided to inhibit the proliferation of olfactory neurons and non-neuronal tissue. The medium was replaced by fresh serum free medium (SFM) twice a week. Nasal explants were fixed for 1 h at 7 days *in vitro* with 4% PFA and processed for immunocytochemistry.

### Electrophysiology

Adult male and female C57BL/6J *GnRH::GFP* homozygous mice were housed under 12-h light/dark cycles (lights on at 0700 hours) with *ad libitum* access to food and water. All experimentation was approved by the University of Otago Animal Welfare and Ethics Committee. The oestrous cycle stage of female mice was determined by daily vaginal smears, and all male, diestrous and proestrous mice killed for experiments between 1000 hours and 1100 hours. Electrophysiology was undertaken on 250-μm-thick coronal brain slices obtained from *GnRH::GFP* mice. Brain slices were incubated for at least 1 h in equilibrated (95% O_2_, 5% CO_2_; 30 °C) aCSF, containing (in mM): 118 NaCl, 3 KCl, 2.5 CaCl_2_, 1.2 MgCl_2_, 11 D-glucose, 10 HEPES, and 25 NaHCO_3_, before being transferred to a submerged recording chamber where they were perfused with aCSF at 2–3 ml min^−1^, maintained at 32±1 °C. Whole-cell or cell-attached recordings of GnRH neurons were undertaken using a fixed-stage upright fluorescence microscope (BX51WI; Olympus) with GFP-tagged GnRH neurons identified briefly using fluorescence and then patched under Nomarski differential interference contrast optics (a × 40 water-immersion objective). Patch pipettes were pulled from glass capillaries (inner diameter, 1.18 mm; outer diameter, 1.5 mm) with a microelectrode puller (Sutter Instruments) and had 1.8–3.5-MΩ resistances when filled with the pipette solution composed of the following (in mm): 135 KCl, 5 NaCl, 0.22 CaCl_2_, 10 HEPES, 1 BAPTA, 2 MgATP, 0.2 Na_2_ATP, 0.2 Na_2_GTP, 7 phosphocreatine-Tris, pH 7.35 adjusted by KOH (∼290 mOsmol). Signals (voltage and current) were amplified with a Multiclamp 700B (CV7B; Molecular Devices) and sampled on-line using a Digidata 1440A interface (Molecular Devices) connected to a personal computer. Signals were filtered (3 or 10 kHz; Bessel filter of Multiclamp 700B) before being digitized at a rate of 1 or 10 kHz. Acquisition and subsequent analysis of the acquired data were performed with the Clampex 10 suite of software (Molecular Devices) and Origin pro 7.5 (OriginLab). AMH (0.04–4 nM) was added to the perifusing (2–3 ml min^−1^) aCSF for 1–3 min. In some experiments, the AAB cocktail (kynurenic acid 2 mM, CNQX 20 μM and GABAzine 5 μM) was included in the perfusion medium before adding AMH. For cell-attached studies, action currents were analysed by determining the number of events per 1 s bin across the time of the recording. A cell was considered to have changed its firing rate if the mean firing frequency in response to AMH (over 2 min) was significantly different from its firing rate in the 2 min control period immediately before testing with AMH (*P*<0.05, paired sample Wilcoxon signed-ranks test). Differences between groups were assessed using the Mann–Whitney U-test. For whole-cell recordings, patch pipettes were filled with an internal solution including (in mM) 128 Kgluconate, 8 KCl, 10 HEPES, 0.4 Na_2_GTP, 4 MgATP, 2 BAPTA (1,2-bis(2-aminophenoxy)ethane-*N*,*N*,*N*′,*N*′-tetra-acetic acid), 0.5 CaCl_2_ and 5 phosphocreatine-Na_2_ (pH 7.43 adjusted by KOH, ∼290 mOsmol). A ramp protocol (from −120 to −20 mV, duration of 1.5 or 3 s) was used before, during and after AMH action to obtain the cell conductance and reversal potential of AMH current. The different doses of recombinant AMH used in this study in the different experimental paradigms, *in vitro* and *in vivo*, are listed in Table 2.

### Prenatal androgen treatment to model PCOS

Adult females were paired with males and checked for copulatory plugs, indicating day 1 of gestation. Pregnant dams were given subcutaneous injections of sesame oil vehicle (200 μl) alone or containing 250 μg of dihydrotestosterone on days 16, 17 and 18 of pregnancy. PNA-treated and prenatally vehicle-treated (control) female offspring were studied in postnatal life.

### Pulsatile LH measurements

Mice were habituated with daily handling for 3–4 weeks. Blood samples (5 μl) were taken from the tail in 10-min intervals for 2 h (between 1200 hours and 1500 hours), diluted in PBS-Tween and immediately frozen. LH levels were determined by sandwich ELISA^37^. A 96-well high-affinity binding microplate (9018; Corning) was coated with 50 μl of capture antibody (monoclonal antibody, anti-bovine LH beta subunit, 518B7; University of California) at a final dilution of 1:1,000 (in 1 × PBS, 1.09 g of Na_2_HPO_4_ (anhydrous), 0.32 g of NaH_2_PO_4_ (anhydrous) and 9 g of NaCl in 1,000 ml of distilled water) and incubated overnight at 4 °C. Wells were incubated with 200 μl of blocking buffer (5% (w/v) skim milk powder in 1 × PBS-T (1 × PBS with 0.05% Tween 20) for 2 h at room temperature. A standard curve was generated using a twofold serial dilution of mLH (reference preparation, AFP-5306A; National Institute of Diabetes and Digestive and Kidney Diseases−National Hormone and Pituitary Program (NIDDK-NHPP)) in 0.2% (w/v) bovine serum albumin−1 × PBS-T. The LH standards and blood samples were incubated with 50 μl of detection antibody (polyclonal antibody, rabbit LH antiserum, AFP240580Rb; NIDDK-NHPP) at a final dilution of 1:10,000 for 1.5 h (at RT). Each well containing bound substrate was incubated with 50 μl of horseradish peroxidase-conjugated antibody (polyclonal goat anti-rabbit; Vector) at a final dilution of 1:10000. After a 1.5-h incubation, 100 μl of *o*-phenylenediamine (002003; Invitrogen), substrate containing 0.1% H_2_O_2_ was added to each well and left at RT for 30 min. The reaction was stopped by the addition of 50 μl of 3 M HCl to each well, and absorbance of each well was read at a wavelength of 490 nm. Pulses were confirmed using DynPeak^67^.

### Statistics

All analyses were performed using Prism 5 (GraphPad Software) and assessed for normality (Shapiro–Wilk test) and variance, when appropriate. Sample sizes were chosen according to standard practice in the field. Data were compared by a two-tailed unpaired Student's *t*-test or one-way analysis of variance for multiple comparisons followed by Fisher's least significant difference *post hoc* test. For comparisons between two groups that did not have a normal distribution, the non-parametric Wilcoxon signed-ranks test was used. The significance level was set at *P*<0.05 in all cases. Data are represented by means±s.e.m. The number of biologically independent experiments, *P* values, age and sex of the animals are indicated in the figure legends.

## Additional information

**How to cite this article:** Cimino, I. *et al.* Novel role for anti-müllerian hormone in the regulation of GnRH neuron excitability and hormone secretion. *Nat. Commun.* 7:10055 doi: 10.1038/ncomms10055 (2016).

## Supplementary Material

Supplementary Information Supplementary Figure 1

## Figures and Tables

**Table 1:** Percentage of GnRH neurons activated by AMH in males and females mice.

	**Responding cells (number)**	**Hz (control)**	**Hz (AMH)**	**% Increase**	**Duration (min)**
**Male**	5 of 9 (56%)	1.03±0.56	2.81±0.50	80±5	17.9±2.9
**Dioestrus**	7 of 11 (64%)	0.41±0.13	2.11±0.43	70±13	15.0±1.2
**Proestrus**	11 of 24 (49%)	0.48±0.16	1.41±0.24	73±7	9.2±1.1**

AMH, anti-Müllerian hormone.

Table showing the number of GnRH cells activated by AMH with % in brackets; the mean firing frequency before and in response to AMH exposure; the percentage increase in firing rate, and duration of increased firing.

**P<0.005 Fisher LSD test, compared with dioestrus. Values shown are means±s.e.m.

**Table 2:** Doses of recombinant AMH used in *ex vivo* and *in vivo* experiments

***Ex vivo***			***In vivo***
**Experiments**	Electrophysiology (brain slices)	Organotypic cultures	i.c.v injections
**Species**	Mouse (C57BL/6J)	Rat (Sprague Dawley)	Mouse (C57BL/6J)
**Methodology**	Patch clamp	ELISA (GnRH)	ELISA (LH)
**[AMH] nM**	0.04–4	125	50–3,000

AMH, anti-Müllerian hormone; ELISA, enzyme-linked immunosorbent assay; LH, luteinizing hormone.
