# Supplementary material for: Novel role for anti-Müllerian hormone in the regulation of GnRH neuron excitability and hormone secretion
Source: Nat Commun. 2016 Jan 12;7:10055. doi: 10.1038/ncomms10055 (PMC4729924; doi:10.1038/ncomms10055)
Supplement: Supplementary Information — Supplementary Figure 1 [file ncomms10055-s1.pdf]

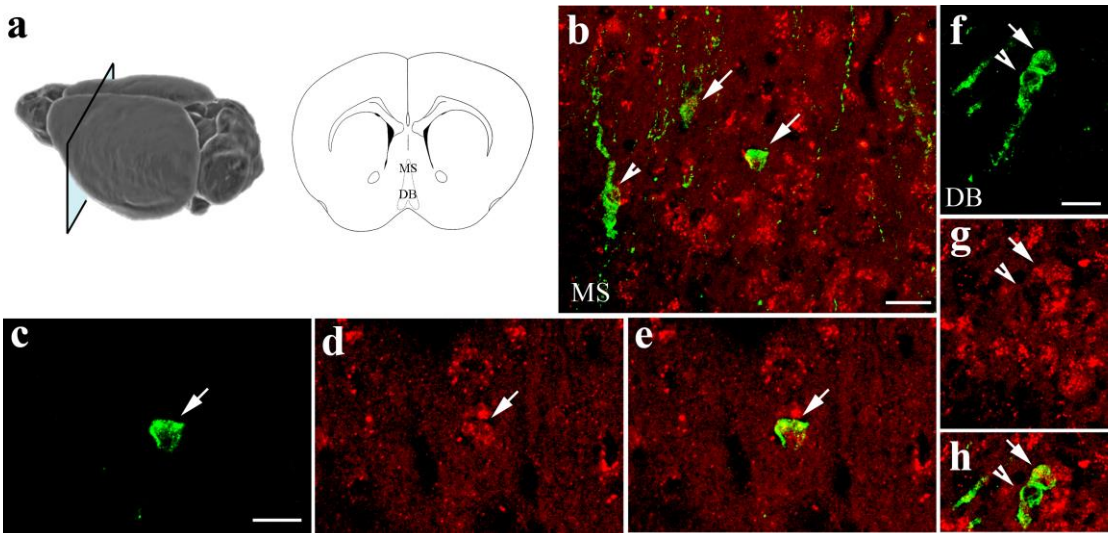

Supplementary Figure 1 *AMHR2 expression in other brain regions containing GnRH cell bodies.* (**a**) Schematic representation of a mouse brain. The coronal section plane contains the medial septum (MS) and the diagonal band of Broca (DB). (**b-h**) Confocal representative photomicrographs showing GnRH (green) and AMHR2 (red) immunoreactivity in coronal sections of adult female mice (P90; number of immunostained brains, n = 3), respectively in the MS (**b-e**) and in the DB (**f-h**). Arrows point to GnRH neurons expressing AMHR2 and arrowheads point to GnRH cells immunonegative for AMHR2. Scale bars, (**b**) 20 µm, (**c-h**) 10 µm.
